# Comparison of physicochemical and *in vitro* hypoglycemic activity of bamboo shoot dietary fibers from different regions of Yunnan

**DOI:** 10.3389/fnut.2022.1102671

**Published:** 2023-01-12

**Authors:** Yufan Dong, Qin Li, Yuhong Guo, Yihe Zhao, Jianxin Cao

**Affiliations:** ^1^Faculty of Food Science and Engineering, Kunming University of Science and Technology, Kunming, China; ^2^Institute of Forestry Industry, Yunnan Academy of Forestry and Grassland, Kunming, China

**Keywords:** bamboo shoot, dietary fiber, physicochemical property, microstructure, hypoglycemic activity

## Abstract

In this study, the physicochemical properties, thermal characteristics, and *in vitro* hypoglycemic activity of dietary fibers extracted from four bamboo shoots were characterized and compared. The results showed that *Dendrocalamus brandisii* Munro (C-BSDF) had the highest dietary fiber content (6.1%) and the smallest particle size (222.21 μm). SEM observations found that C-BSDF exhibited a loose and porous microstructure, while FTIR and XRD confirmed that C-BSDF had a higher degree of decomposition of insoluble dietary fiber components and the highest crystallinity, resulting in a better microstructure. Furthermore, C-BSDF exhibited excellent physiochemical properties with the highest water hold capacity, water swelling capacity, and preferable oil holding capacity. Thermal analysis showed that C-BSDF had the lowest mass loss (64.25%) and the highest denaturation temperature (114.03°C). The hypoglycemic activity of dietary fibers from bamboo shoots were examined *in vitro* and followed this order of activity: C-BSDF>D−BSDF>A−BSDF>B-BSDF. The inhibition ratios of GAC, GDRI and α-amylase activity of C-BSDF were 21.57 mmol/g, 24.1, and 23.34%, respectively. In short, C-BSDF display excellent physicochemical and functional properties due to its high soluble dietary fiber content, small particle size with a high specific surface area, and loose microstructure. Thus, *D. brandisii* Munro can be considered a promising new source of dietary fiber for hypoglycemic health products.

## 1. Introduction

Dietary fibers have many physiological functions, such as lowering blood sugar and fat levels, inducing weight loss, regulating intestinal microorganisms, and preventing diabetes, and intestinal cancer (1, 2). These fibers are known as the “seventh nutrient” of human beings, and their role in maintaining body weight and regulating health has attracted increasing attention. Studies have found that consuming large quantities of dietary fibers can play a positive role in human health (3). Studies have shown that dietary fibers can reduce the risk of type 2 diabetes by reducing energy intake and controlling glycemia. Moreover, fibers can also reduce blood glucose fluctuations and reduce insulin response (4). Blood glucose control is mainly affected by delayed gastric emptying, small intestinal transit time, and digestion and absorption of large amounts of nutrients (5). Indeed, dietary fibers have been widely used in developing and utilizing health care products.

Plants are an important source of dietary fiber and are often characterized by high yield and low utilization rates. Plant dietary fibers mainly include cellulose, hemicellulose, lignin and galactomannan, derived from plant roots, stems (6), leaves (7), fruits (8, 9), and buds (10). The physicochemical properties of these fibers, such as their water-holding and oil-holding capacities have been widely investigated by numerous researchers. Moreover, their functional properties, such as reducing blood sugar and blood fat, improving intestinal flora, preventing constipation and colon cancer have been explored to clarify the mechanisms through which dietary fibers exert their beneficial effects. Therefore, there has been increasing interest over recent years in developing additional dietary fiber resources.

The use of natural sources of bioactive compounds, especially substances extracted from plants, for food fortification has attracted much attention (11, 12). Bamboo shoots are nutritious forest food, rich in dietary fiber, protein, vitamins, phenolic compounds, and phytosterols (13). The Bamboo shoot dietary fiber (BSDF) os mainly composed of cellulose, hemicellulose, lignin, and polysaccharides. It has better water and oil retention than other dietary fibers (14). However, the properties of BSDF vary in different regions, while many the physicochemical and functional properties of many BSDF remain unexplored. More importantly, the mechanism behind the differences in physicochemical properties and functions of different types of BSDF is still unclear.

The Yunnan province is located in southwest China, at the junction of three very different natural geographical regions (15). Yunnan creates favorable conditions for the collection, diffusion and differentiation of bamboo plants due to its unique environment. Indeed, it has become a region with abundant bamboo species, ecological types and natural bamboo forests, containing cold- and hot-temperature bamboo forests in high-altitude areas. The bamboo resources in Yunnan are characterized by a wide variety of bamboo shoots of good quality. Bamboo shoot resources development prospect is broad and their varieties have an irreplaceable market competitive advantage. Among the numerous bamboo species in Yunnan, more than half of them can be used as bamboo shoots and many are high-quality varieties unique to Yunnan or rare in other places, which are superior to other parts of the world in terms of quantity and quality (16). Therefore, it is necessary to study representative bamboo shoots in different regions of Yunnan, especially with regard to the physical and functional properties of BSDF to provide useful information for the development of high-quality bamboo shoot by-products in Yunnan.

Dietary fiber extracted from different sources has a different chemical compositions, structures, and functional properties (17). In this study, we aimed to evaluate the physicochemical and structural characteristics, as well as the hypoglycemic activity of different BSDF. First, BSDF were extracted from four bamboo shoots collected from four different regions of the Yunnan Province. Then, the composition, physicochemical properties, and microstructure of four different BSDF samples were analyzed. Finally, their hypoglycemic activity *in vitro*, including glucose adsorption capacity (GAC), α-Amylase activity inhibition ratio (α-AAIR), and glucose dialysis retardation index (GDRI), were compared. The results of this study can provide a scientific basis for the development of bamboo shoot products for food and nutrition applications.

## 2. Materials and methods

### 2.1. Materials

**FIGURE 1:**
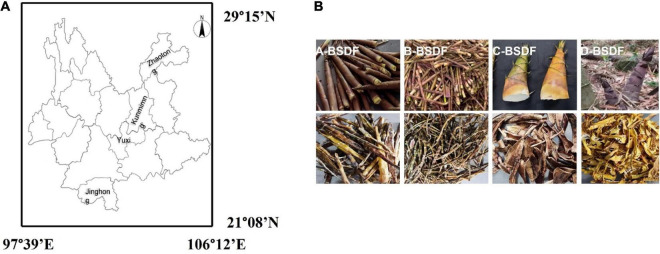
Geographical locations of different bamboo shoot samples in Yunnan province of China **(A)**, extraction process of bamboo shoot dietary fiber (BSDF) **(B)**.

Four kinds of bamboo shoots with different families were collected from different regions of the Yunnan province, China (Figure 1A). *Dendrocalamus brandisii* Munro was collected from the Jinghong Dai Autonomous Prefecture of Xishuangbanna, *Phyllostachy sulphurea* was collected from the Yunnan Kunming Academy of Forestry and Grassland Sciences Arboretum, *Qiongzhuea tumidinoda* was collected from the Daguan County of Zhaotong, *Pleioblastus amarus* Keng was collected from the Xinping Yi Autonomous County.

Sodium hydroxide, ethanol, phosphoric acid, sulfuric acid, hydrochloric acid, and boric acid were analytically purchased from Zhiyuan Chemical Reagent Co., Ltd. (Tianjin, China). Soybean oil and potato starch were purchased from local supermarkets at Kunming. The glucose detection kit was supplied by Solarbio Technology Co., Ltd. (Beijing, China). Protease, α-amylase, and glucosidase were obtained from Yingxin Laboratory Equipment Co., Ltd. (Shanghai, China).

### 2.2. Dietary fiber extraction procedure

Bamboo shoot dietary fiber was extracted according to the method of Zhang et al. (17) with modifications. Bamboo shoots were put into an electrothermal drying oven to dry, were crushed with a pulverizer, and passed through a 40-mesh sieve. Bamboo shoot powder (100 g) was soaked in 1 L 0.5 mol/L NaOH and heated at 50°C for 1 h in a water bath. After cooling to room temperature, the precipitate was collected by centrifugation at 6,800 *g* for 30 min to obtain insoluble dietary fiber. The supernatant was precipitated with four times the volume of 95% ethanol and allowed to stand at room temperature for 1 h. Then the suspension was centrifuged at 6,800 *g* for 15 min, and the precipitate was collected as soluble dietary fiber. Soluble and insoluble dietary fibers were mixed evenly and freeze-dried. The dietary fiber of *P. sulphurea*, *Q. tumidinoda*, *D. brandisii* Munro, and *P. amarus* Keng were named A-BSDF, B-BSDF, C-BSDF, and D-BSDF, respectively.

### 2.3. Composition analysis of extracted dietary fibers

The composition and content of BSDF were determined according to the following methods. Moisture and ash contents were determined using the AOAC method 925.40 (2005): samples were dried at 105°C (moisture) and 500°C (ash), respectively, until a constant weight was achieved. Crude protein content was determined by the Kjeldahl method. The nitrogen conversion factor was 6.25 according to the AOAC method 955.04 (2000). The crude fat content of fiber samples were estimated by Soxhlet extraction with petroleum ether as solvent according to the AOAC method 920.39 (2005). The contents of soluble dietary fiber and insoluble dietary fiber were determined by the AOAC method 991.43 (1994) (18), and the amount of total dietary fiber was calculated.

### 2.4. Particle size analysis

Particles were measured using a laser particle size analyzer (Mastersizer 2000, UK) and the method described by Wang et al. (19). The precipitation of different varieties of BSDF samples was adjusted to 0.01% (m/v) and tested at an ambient temperature of 25°C. The refractive indices of the dispersant and the sample were 1.33 and 1.53, respectively, and the shading parameter was 1–2%. The particle size distribution is represented as d0.1 (μm), d0.5 (μm), d0.9 (μm), and the Sauter mean diameter is represented as D [4,3] (μm) and D [3,2] (μm).

### 2.5. Scanning electron microscopy (SEM)

The microstructure of different BSDF was observed by a TM3030Plus SEM (Hitachi, Tokyo, Japan) at an accelerating voltage of 15.0 kV (20). A double-sided adhesive was adhered to the cylindrical aluminum table, and the sample was evenly coated on it. Subsequently, the sample was placed in the instrument for gold plating (10 min, 2 mbar). Finally, the surface morphology of the sample was observed. The picture scanning multiple was 200 ×.

### 2.6. Fourier transform infrared spectroscopy (FTIR)

Samples were analyzed using a fourier transform infrared spectroscope (FTIR, Thermo Scientific Nicolet iS50) as previously reported by Jiang et al. (21). The sample was ground with KBr (1:100, w/w) and pressed into slices. Then, the spectrum in the range of 4,000–400 cm^–1^ was obtained with a resolution of 1 cm^–1^. The chemical structure of each BSDF was obtained.

### 2.7. X-ray diffraction (XRD)

The crystalline region of BSDF was observed by X-ray diffractometer (X’Pert PRO MPD, PANalytical BV., the Netherlands). The specific conditions were set as: tube voltage of 40 kV, incident current of 40 mA and scan range of 5–70°. The step size and scan rate were 0.05 and 0.21°/s, respectively. The MDI Jade 5 software (Materials Data, Inc., Livermore, CA, USA) was employed to calculate the peak area and crystallinity of the dietary fiber (22) using the following formula:


(1) $$D\,C\%=\frac{A_{C}}{A_{c}+A_{a}}\times 100$$

Where *Ac* is the area of the crystalline region in the XRD pattern, and *Aa* is the area of the amorphous region in the XRD pattern.

### 2.8. Chemical characteristics

#### 2.8.1. Thermogravimetric analysis (TGA)

Thermogravimetric analysis of fiber samples was carried out using (DSC/TGA Discovery SDT 650). The sample (10 mg) was heated from 20 to 800°C at a speed of 10°C/min.

#### 2.8.2. Differential scanning calorimetry (DSC)

Samples were analyzed for differential scanning calorimetry as previously reported by Wen et al. (23). First, 10 mg samples were weighed and placed in a high-purity alumina crucible and the temperature of the thermo gravimetric analyzer was adjusted to 23∼600°C. The temperature was raised at a rate of 10°C/min under nitrogen protection. The flow rate of nitrogen was 100 mL/min during continuous heating.

#### 2.8.3. Color measurement

The chromaticity was conducted according to the procedure used by Felisberto et al. (24). It was composed of: brightness (L*, where L* = 0 is black, L* = 100 is white) and redness (a*, where a* > 0 indicates red, a* < 0 means green) and yellowness (b*, where b* > 0 indicates yellow, b* < 0 indicates blue).

### 2.9. Physical properties

#### 2.9.1. Water holding capacity

The water holding capacity was determined according to the method of Du et al. (25). Each BSDF sample was weighed (1 g) and mixed evenly in 30 mL of deionized water. Samples was stirred at room temperature for 24 h, centrifuged at 4,000 *g* for 15 min, and the pellet dried in an oven at 105°C. WHC was calculated according to the following equation:


(2) $$W\,H\,C/\left(g\cdot g^{-1}\right)=\frac{m_{2}-m_{1}}{m_{1}}$$

Where *m*_2_ is the weight of sample residue containing water after centrifugation (*g*); *m*_1_ is the weight (*g*) of the sample dried to constant weight.

#### 2.9.2. Water swelling capacity

The water swelling capacity was determined by the method described by Huang et al. (26). Each BSDF sample was weighed (1 g), mixed with 20 mL of deionized water in a 25 mL graduated test tube and allowed to stand at room temperature for 20 h. The expansion volume of the sample was recorded as follows:


(3) $$W\,S\,C/\left(m\,l\cdot g^{-1}\right)=\frac{v_{2}-v_{1}}{m_{0}}$$

Where *v*_2_ is the volume (mL) of the sample after water absorption; *v*_1_ is the volume of the sample before water absorption (mL); *m*_0_ is the mass of the sample before water absorption (*g*).

#### 2.9.3. Oil holding capacity

The oil holding capacity was determined according to the method of Yu et al. (27). Each BSDF sample was weighed (0.5 g), mixed with 30 mL soybean oil in a centrifuge tube, and allowed to stand at room temperature for 20 h. After centrifugation at 4,000 *g* for 15 min, the upper layer of oil was poured out and OHC was calculated as follows:


(4) $$O\,H\,C/\left(g\cdot g^{-1}\right)=\frac{m_{4}-m_{3}}{m_{3}}$$

Where *m*_4_ is the mass (*g*) after oil absorption saturation; *m*_3_ is the mass before oil absorption (*g*).

### 2.10. *In vitro* hypoglycemic activity

#### 2.10.1. Glucose adsorption capacity (GAC)

Glucose adsorption capacity (GAC) was determined according to the method of Ma et al. (22). Each BSDF sample was weighed (1 g), mixed with 100 mL of 100 mmol/L glucose solution and water bathed at 37°C for 6 h. Samples were centrifuged at 5,000 *g* for 15 min. GAC represents the millimolar number of glucose retained per gram of BSDF:


(5) $$GAC\;mmol.g^{-1}=\frac{(c_{0}-c_{1}).v}{w}$$

Where *c*_0_ is the glucose concentration (mmol/L) in the original solution before adsorption; *c*_1_ is the concentration of glucose in the supernatant (mmol/L) after adsorption equilibrium; *v* is the supernatant liquid volume (L); *w* is the weight of BSDF sample (g).

#### 2.10.2. Glucose dialysis retardation index (GDRI)

The glucose dialysis retardation index (GDRI) was prepared according to the procedure used by López et al. (28). Each BSDF sample was weighed (0.5 g), put into a 15 mL 100 mmol/L glucose solution and fully mixed. The mixed solution was added to a dialysis bag with 14,000 Da molecules. Then the mixture was dialyzed with 200 mL distilled water and placed in a 37°C thermostatically controlled water bath. After 30, 60, 90, and 120 min, 1 mL of dialysate was collected and its glucose content was measured. A control experiment was conducted without adding BSDF. GDRI is calculated as follows:


(6) $$G\,D\,R\,I\%=100-\frac{c_{2}}{c_{3}}\times 100$$

Where *c*_2_ is the glucose diffused from the sample; *c*_3_ is glucose diffused from the control group.

#### 2.10.3. α–amylase activity inhibition ratio (α-AAIR)

The determination of α-amylase activity inhibition ratio (α-AAIR) referred to the method of Benitez et al. (29). Each BSDF sample was weighed (1 g), mixed with 4.0 mg α-amylase (40 U/mg) and 40 mL 4% starch solution, and incubated at 37°C for 1 h. Then, BSDF samples were centrifuged at 4,000 *g* for 20 min, and the glucose content in the supernatant was measured using a glucose detection kit. A control experiment was conducted without adding BSDF. α-AAIR is calculated using the following formula:


(7) $$\alpha -AAIR\left(\%\right)=\frac{\left(C_{4}-C_{5}\right)}{C_{4}}\times 100$$

Where *c*_4_ is the control glucose concentration; *c*_5_ is the glucose concentration of the BSDF sample.

## 3. Results and discussion

### 3.1. Composition and content analyses of different bamboo shoot dietary fibers

Basic components of BSDF extracted from *P. sulphurea, Q. tumidinoda, D. brandisii* Munro, and *P. amarus* keng are shown in Table 1. The protein content of extracted BSDF was significantly different across the bamboo varieties studied. There was no significant difference in fat content, except in D-BSDF. The ash content of different varieties of BSDF was classified from lowest to highest: C-BSDF < A − BSDF<D−<B-BSDF, in which the lowest value was 7.14% for C-BSDF. The moisture content of BSDF from highest to lowest was: A-BSDF> D−BSDF>B−BSDF>C-BSDF, in which the highest value was 13.53% ± 0.11% for A-BSDF. The highest protein content was found in C-BSDF (16.12% ± 0.18%), which was 1.93 times that of D-BSDF–the fat content of D-BSDF was 0.20% ± 0.01%. C-BSDF had the highest soluble dietary fiber content (6.1 % ± 0.19 %), followed by D-BSDF, B-BSDF, and A-BSDF, which were higher than values found for walnut powder (0.88–6.53%) (30) and rice bran (1.5%) (23). There was no significant difference between other varieties.

**TABLE 1:** Basal composition and content of different bamboo shoots.

Basal component (%)	A-BSDF	B-BSDF	C-BSDF	D-BSDF
Ash	10.92 ± 0.25^b^	11.78 ± 0.16^c^	7.14 ± 0.08^a^	11.07 ± 0.44^b^
Moisture	13.53 ± 0.11^c^	11.26 ± 0.81^a^	11.17 ± 0.12^a^	12.35 ± 0.42^b^
Protein	10.70 ± 0.09^b^	12.60 ± 0.34^c^	16.12 ± 0.18^d^	8.33 ± 0.09^a^
Fat	0.14 ± 0.03^a^	0.13 ± 0.01^a^	0.13 ± 0.02^a^	0.20 ± 0.01^b^
Total dietary fiber	63.91 ± 0.76^a^	63.64 ± 1.24^a^	64.97 ± 0.89^a^	67.6 ± 0.79^a^
Insoluble dietary fiber	59.57 ± 0.98^a^^b^	60.01 ± 1.13^a^^b^	58.84 ± 0.08^a^	61.82 ± 0.5^b^
Soluble dietary fiber	4.34 ± 0.07^b^	4.03 ± 0.06^a^	6.13 ± 0.19^d^	5.78 ± 0.07^c^

Different lowercase letters in the same row indicate significant differences between groups (*p* < 0.05).

### 3.2. Particle size distribution of dietary fiber from different bamboo shoots

**FIGURE 2:**
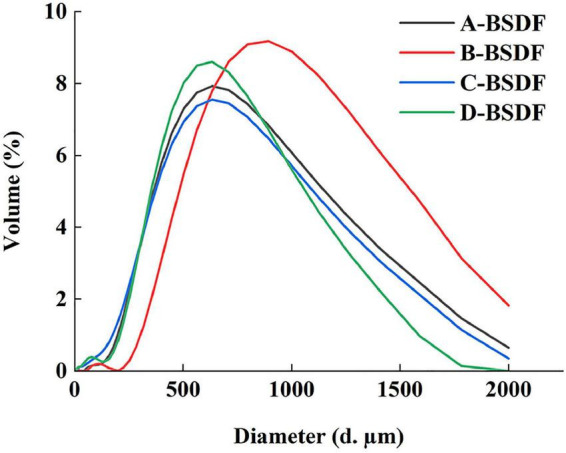
Size distribution chart.

The particle size distribution is shown in Table 2 and Figure 2. The peak shape of A-BSDF, C-BSDF, and D-BSDF was sharper than that of B-BSDF (Figure 2), which indicated that the particle size distribution of A-BSDF, C-BSDF, and D-BSDF was more concentrated and uniform. Compared with B-BSDF, the main peak of A-BSDF, C-BSDF and D-BSDF shifted to the left and the particle size decreased. Table 2 shows the d_0.1_, d_0.5_, d_0.9_, D [4,3], and D [3,2] of BSDF. The d_0.1_, d_0.5_, and d_0.9_ represent the diameters corresponding to 10, 50, and 90% of the cumulative particle size distribution, respectively. D [4,3] and D [3,2] are the volume and surface area average diameter, respectively.

**TABLE 2:** Particle size distribution of different varieties of bamboo shoot dietary fibers.

Samples	Particle size distribution (μ m)				
	**d_0.1_ (μm)**	**d_0.5_ (μm)**	**d_0.9_ (μm)**	**D [4,3] (μm)**	**D [3,2] (μm)**
A-BSDF	279.03 ± 3.91^c^	593.39 ± 14.62^b^	1190.42 ± 42^b^	670.84 ± 18.85^b^	478.37 ± 10.12^b^
B-BSDF	413.10 ± 6.48^d^	788.45 ± 7.22^c^	1406.41 ± 5.38^c^	851.97 ± 6.40^c^	627.76 ± 51.41^c^
C-BSDF	222.21 ± 7.35^a^	560.29 ± 23.62^a^^b^	1140.53 ± 24.40^b^	626.70 ± 18.95^a^	309.17 ± 10.92^a^
D-BSDF	249.22 ± 12.49^b^	548.20 ± 22.42^a^	1026.10 ± 60.68^a^	593.95 ± 29.73^a^	300.90 ± 16.99^a^

Different lowercase letters in the same row indicate significant differences between groups (*p* < 0.05).

The d_0.1_, d_0.5_, d_0.9_, D [4,3], and D [3,2] of C-BSDF and D-BSDF were lower than those of A-BSDF and B-BSDF, especially d_0.1_, D [4,3], and D [3,2], indicating that C-BSDF and D-BSDF have the smallest particle size among all samples. As depicted in Table 2, the difference between D [4,3] and D [3,2] of C-BSDF was the largest, indicating that the specific surface area of C-BSDF was the largest among groups. Dietary fiber has a larger specific surface area, which may yield a better effect on physical and chemical properties, such as WHC, OHC, and swelling capacity (26). Therefore, *D. brandisii* Munro or *P. amarus* keng have the potential to be candidates for obtaining high-quality dietary fibers for functional foods.

### 3.3. Structure characterization of different bamboo shoot dietary fibers

#### 3.3.1. Microstructure of different bamboo shoot dietary fibers

**FIGURE 3:**
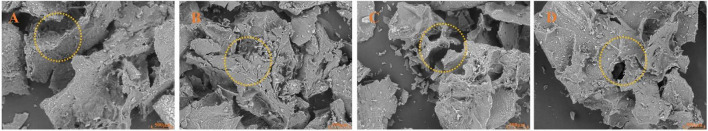
Scanning electron microscopy images (200x) of dietary fiber from different bamboo shoots. A-BSDF **(A)**, B-BSDF **(B)**, C-BSDF **(C)**, D-BSDF **(D)**.

The microstructure of dietary fiber is related to its pore characteristics and effective surface properties (31). The surface morphological characteristics of different varieties of BSDFs are shown in Figure 3. All samples showed irregular sheet distribution. It was obvious that there were many cracks and holes on the surface of the fibers. This may be because the sodium hydroxide solution used in the chemical extraction process destroyed the network structure of dietary fiber (32). Compared with other BSDFs, C-BSDF, which had the smallest particle size, also had a more porous and looser microstructure. The loose structure of dietary fiber may expose more groups, which is beneficial to its physicochemical properties. These results showed that different sources of origin may induce different functional properties of BSDF.

#### 3.3.2. FTIR analysis of different bamboo shoot dietary fibers

**FIGURE 4:**
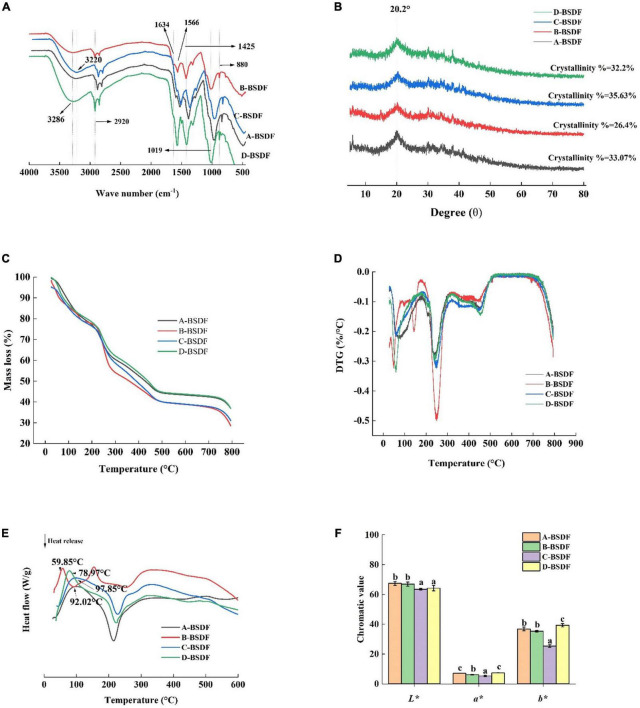
FT-I R of DF from different bamboo shoots **(A)**, X-ray diffraction of dietary fiber from different bamboo shoots **(B)**, TG of DF from different bamboo shoots **(C)**, DTG of DF from different bamboo shoots **(D)**, DSC of DF from different bamboo shoots **(E)**, color index of dietary fiber in different bamboo shoots **(F)**.

Fourier transform infrared spectroscopy provides the group composition of the BSDF structure. Here we used FTIR to analyze BSDF prepared from different varieties of bamboo shoots and the results are shown in Figure 4A. The spectral profile and peak shape of all BSDFs were similar, while the chemical composition was the same. However, the absorbance and wavenumber of some characteristic bands changed.

All BSDFs showed a broad absorption peak at about 3,286 cm^–1^, which can be attributed to the stretching vibration of -OH bond (21). Compared with other BSDFs, a blue shift of B-BSDF from 3,286 to 3,327 cm^–1^ was observed, which may be due to different degrees of hydrogen bond damage caused by the small specific surface area of B-BSDF (33). The wave number of C-BSDF decreased from 3,286 to 3,320 cm^–1^, and a red shift occurred. This may be because the structure of this dietary fiber was changed to some extent and the molecule was more stable (34, 35). This may be the reason for the formation of hydrogen bonds (36). At the same time, the absorption peak intensity of C-BSDF and D-BSDF at 3,286 cm^–1^ were stronger than those of A-BSDF and B-BSDF, indicating that there are more intramolecular hydrogen bonds between hemicelluloses.

The weak absorption band at 2,920 cm^–1^ originates from the vibration of the C-H group, indicating the existence of the typical structure of polysaccharide compounds. The peak near 1,634 cm^–1^ is related to the characteristic absorption of the C = O bond of uronic acid, indicating that samples contained uronic acid, while the weak characteristic peak may be because sodium hydroxide destroys the structure of uronic acid (37). The peak near 1,425 cm^–1^ is related to the vibration of COO-, while the peak at 1,566 cm^–1^ is the characteristic absorption peak of lignin in hemicellulose (38). In addition, the wavelength below 1,300 cm^–1^ is called the fingerprint region, where 950–1,200 cm^–1^ is the characteristic region of carbohydrates, 1,019 cm^–1^ is the vibration of oxygen-containing functional groups of C-O-C carbon skeleton (37). The peak located at about 880 cm^–1^ is related to the deformation vibration of β-CH of the β-glycosidic bond. The lower C-BSDF peak intensity at 880 cm^–1^ may be related to hemicellulose decomposition, exposing more dipole forms, which may result in a high hydration capacity (35). The change of characteristic peaks in C-BSDF may be related to increased water-soluble polysaccharides, which may contribute to the enhancement of functional properties (39).

#### 3.3.3. XRD analysis of different bamboo shoot dietary fibers

The XRD patterns of A-BSDF, B-BSDF, C-BSDF, and D-BSDF are shown in Figure 4B. BSDF has a regular wide peak between 16 and 23°, indicating that there are amorphous or cellulose I crystals and amorphous structures in the crystal region of BSDF (17). The BSDF peaks of 14.98 and 24.44° disappeared, which may be attributed to the denaturation of cellulose by NaOH (40). In addition, the crystallinity of A-BSDF, B-BSDF, C-BSDF and D-BSDF were 33.07, 26.4, 35.63, and 32.2% respectively. The crystallinity of C-BSDF was slightly higher than that of other BSDFs. These results showed that the crystal structure of C-BSDF was stable, which was consistent with FTIR (Figure 4A).

### 3.4. Physicochemical properties of different bamboo shoot dietary fibers

#### 3.4.1. Thermal characteristic analysis of different bamboo shoot dietary fibers

Thermogravimetric analysis results are shown in Figures 4C, D. The distribution process of pyrolysis products between gas and solid phases can be investigated by analyzing the curve trend. In Figures 3C, D, with the increase in temperature, BSDF had two obvious weight loss processes. The first weight loss process occurred at 25–150°C, which is due to dehydration. There was a rapid curve decrease, and the mass loss of BSDF exceeded 10%. The second weight loss process occurred at 215–450°C, which can be attributed to the decomposition of organic compounds (41). In this range, a significant peak appeared on the DTG curve (Figure 4D) near 250°C, indicating that the weight loss rate of the four samples was faster, which may be related to the pyrolysis of hemicellulose and soluble pectin, or the pre-carbonization process of cellulose (17). It can be clearly seen that the peak intensity of B-BSDF was the largest and its weight loss was the fastest. After 450°C there is a slow decomposition process, mainly involved in substances difficult to pyrolyze, such as lignin and other compounds, which is a thermal weight loss process that produces ash and other residues (42). According to Figure 4C, among the four BSDFs, C-BSDF showed the lowest mass loss (64.25%), followed by A-BSDF (65.03%), D-BSDF (66.31%), and B-BSDF (69.88%). The residual mass of C-BSDF was higher than that of A-BSDF, B-BSDF and D-BSDF, indicating that its thermal stability was better, which may be related to its high crystallinity of 35.63% (10) (Figure 4B).

In general, the mass change of the sample during thermal decomposition can be investigated by analyzing the TGA curve, and the energy change of the sample during thermal decomposition can be investigated by analyzing the differential scanning calorimetry (DSC) curve. Mass and energy changes often co-exist in the thermal decomposition process of the sample. However, crystal transformation and melting induce energy, but not mass, change. Therefore, more comprehensive information can be obtained by examining the DSC curve (43). It can be seen from the DSC curve (Figure 4E) that all samples had an obvious endothermic peak near 100°C, which may be due to water evaporation (38), and the endothermic peak transition range was 25–150°C. The endothermic peak of A-BSDF, B-BSDF, C-BSDF and D-BSDF were approximately 104.38, 102.73, 114.03 and 107.09°C, respectively, which were consistent with the results reported by Slavov et al. (44). C-BSDF showed the highest denaturation temperature at 114.03°C due to its high crystallinity (10) (Figure 4B), which indicated C-BSDF was relatively more stable. In the range of 220–270°C, an exothermic peak was observed, which is mainly caused by the oxidation of the molecular side chain (45, 46). In addition, FTIR results (Figure 4A) corroborate that C-BSDF contained more hydrogen bonds, thus more energy was needed to destroy its crystal structure.

Altogether, C-BSDF had smaller particles and showed better thermal stability than other BSDF.

#### 3.4.2. Chromaticity analysis of dietary fibers from different bamboo shoots

Color is one of the main characteristics of food, and it is also the first evaluation index that consumers can get. Strong colors are widely used in food, and the application prospect is wide. The L*, a*, and b* of different varieties of BSDF are shown in Figure 4F. The values of L*, a*, and b* represent the brightness, redness, and yellowness of BSDF, respectively. The highest a* and b* values were found in D-BSDF, while the lowest were found in C-BSDF. L* of C-BSDF and D-BSDF were significantly lower than that of A-BSDF and B-BSDF. These results showed that the color of D-BSDF was darker than the other three bamboo shoots. Therefore, the use of D-BSDF in the animal food industry (including pet food) can increase the richness of food color and promote animal appetite and feed intake.

#### 3.4.3. Water holding capacity

**FIGURE 5:**
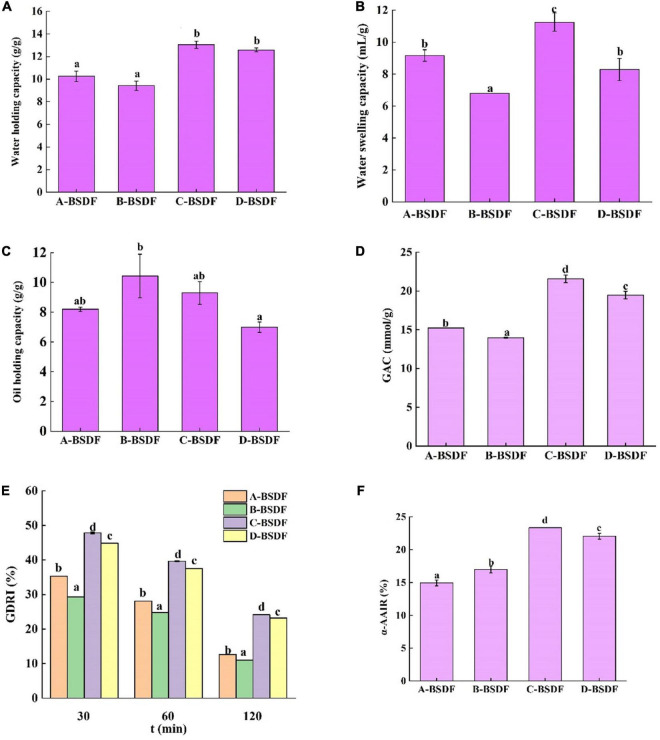
Water holding capacity of dietary fiber in different bamboo shoots **(A)**, water swelling capacity of dietary fiber in different bamboo shoots **(B)**, and oil holding capacity of dietary fiber i n different bamboo shoots **(C)**, GAC **(D)**, GDRI **(E)**, α-AAIR **(F)** of dietary fiber from different bamboo shoots. Different letters represent significant differences (*p* < 0.05).

Evaluating the water holding capacity of dietary fibers is of great significance in maintaining human health. Increased the stronger the water holding capacity of dietary fibers means that a larger volume of feces is discharged after eating these fibers. Since dietary fibers can induce the penetration of intestinal microorganisms in food residues for fermentation, they reduce rectal and urinary system pressure and prevent constipation and colon cancer (47). As shown in Figure 5A, the water holding capacities of C-BSDF and D-BSDF were 13.04 and 12.58 g/g, respectively, which were higher than those of A-BSDF and B-BSDF. Such higher water holding capacity of C-BSDF and D-BSDF may be attributed to their smaller particle size and larger specific surface area (Table 2). These results are similar to those of Wuttipalakorn et al. (48). In summary, although the water holding capacity of extracted fiber samples was significantly different, it was higher than that of defatted cumin dietary fiber (7.28 g/g) (22), bamboo shoot shell dietary fiber (8.27 g/g) (49), and pear pomace (5.77 g/g) (8).

#### 3.4.4. Water swelling capacity

The swelling capacity refers to the ratio of the volume of dietary fiber to the actual weight of dietary fiber after immersion in water. Dietary fibers may interact with water through two mechanisms: water in the capillary structure due to the surface tension strength, and through the formation of hydrogen bonds and dipoles (26). Figure 5B summarizes the swelling capacity of four varieties of BSDF. Compared with other BSDF samples, the specific surface area of C-BSDF was larger, because it had smaller particles, ultimately resulting in more hydrophilic groups exposed and increased swelling capacity (50). Meanwhile, the swelling capacity of B-BSDF was the lowest, which was parallel with its water holding capacity.

#### 3.4.5. Oil holding capacity

The ability of dietary fiber to retain oil is important for food applications. For example, dietary fibers with high oil holding capacity can reduce oil loss and absorb or bind cholesterol and bile acids during food processing, which helps reduce blood cholesterol (51). Figure 5C summarizes the oil holding capacity of four varieties of BSDF. The four BSDFs can be arranged as follows: B-BSDF >C−BSDF>A-BSDF>D-BSDF. In particular, B-BSDF had the strongest oil holding capacity (10.42 g/g), which was significantly higher than that of D-BSDF. It was reported that oil holding capacity is related to surface characteristics, hydrophobicity and total charge density of fiber particles (52). Interestingly, the oil holding capacity of all samples was higher than that of pear residue dietary fiber (2.77 g/g) (8), enzymatic modified potato powder (2.89 g/g) (6), bamboo shoot shell dietary fiber (5.79 g/g) (49), and Maca residue fiber (5.79 g/g) (53).

### 3.5. Effects of different varieties of bamboo shoot dietary fibers on *in vitro* hypoglycemic activity

#### 3.5.1. Glucose adsorption capacity (GAC)

Previous studies have shown that dietary fiber from different sources adsorbs glucose in a dose-dependent manner (51). As shown in Figure 5D, the ability of four BSDFs to adsorb glucose can be ranked as: C-BSDF (21.57 mmol/g) > D-BSDF (19.48 mmol/g) > A-BSDF (15.24 mmol/g) > B-BSDF (13.97 mmol/g). The results showed that C-BSDF had a stronger hypoglycemic effect *in vitro*, which may be due to its small particle size and large specific surface area, resulting in an increased glucose adsorption (53). This is beneficial to select sources of high-quality BSDF that enhance the absorption of glucose during gastrointestinal transport, and inhibit hyperglycemia.

#### 3.5.2. Glucose dialysis retardation index (GDRI)

The glucose retardation index is an important indicator to predict the reduction and delay of gastrointestinal glucose absorption of dietary fibers. As shown in Figure 5E, the maximum GDRI value of BSDFs appears at 30 min. The retardation of glucose molecules by fiber particles and the retention of glucose molecules in the fiber network may explain the delay in glucose diffusion (28). The results showed that the GDRI values of different varieties of BSDF were significantly different, which may be due to different particle sizes and the amount of SDF. C-BSDF showed a relatively high GDRI value likely because of its improved delay effect on glucose diffusion.

#### 3.5.3. α–amylase activity inhibition ratio (α-AAIR)

α-amylase is a major enzyme in the process of starch digestion. It helps starch digestion and can cause postprandial hyperglycemia in diabetic patients. Inhibition of α-amylase can hinder the hydrolysis and digestion of carbohydrates in food, reduce the digestion of sugars and effectively control postprandial hyperglycemia. The inhibitory effect of four BSDFs on α-amylase is shown in Figure 5F. There were significant differences, in which C-BSDF showed the greatest *α-AAIR* probably due to its high dietary fiber content (Table 1) and increased specific surface area (29).

## 4. Conclusion

In this study, four different bamboo shoots were used to extract their dietary fiber, and analyze their physicochemical properties, structural characteristics, and *in vitro* hypoglycemic activity. Compared with A-BSDF and B-BSDF, C-BSDF showed the highest dietary fiber content, smallest particle size with a large specific surface area, and a porous and loose microstructure. This is consistent with FTIR and XRD data. Therefore, compared with other BSDFs, C-BSDF exhibited the best physiochemical properties, including thermal characteristics, water holding capacity, water swelling capacity and oil holding capacity, that translated into increased glucose adsorption and delayed diffusion.

Postprandial hyperglycemia is one of the main symptoms of type II patients. Strict control of blood glucose levels, especially postprandial blood glucose levels, is an effective measure to delay disease progression. Therefore, our findings unveil the potential hypoglycemic mechanism of BSDF and provide valuable information for exploring high-quality sources of BSDF. *D. brandisii* Munro may be a promising variety to obtain high-quality BSDF and has the potential to be added to food and hypoglycemic health products as a functional component.

## Data availability statement

The raw data supporting the conclusions of this article will be made available by the authors, without undue reservation.

## Author contributions

YD and YZ conceived and designed the methods and framework. YD analyzed the data, wrote the manuscript, contributed to the interpretation of the data, discussion of results, and writing of the manuscript. QL and YG contributed to the data collection and data analysis. JC contributed to the data collection, interpretation of the data, and discussion of results. All authors read and approved the final manuscript.
